# Crystal structure and computational study of 3,4-dihy­droxy-3-hy­droxy­methyl-9-methyl-6-methyl­idene-3a,4,5,6,6a,9,9a,9b-octa­hydro­azuleno[4,5-*b*]furan-2,8(3*H*,7*H*)-dione

**DOI:** 10.1107/S2056989015019623

**Published:** 2015-11-04

**Authors:** Ísmail Çelik, Mehmet Akkurt, Hüseyin Akşit, Ramazan Erenler, Santiago García-Granda

**Affiliations:** a Department of Physics, Faculty of Arts and Sciences, Cumhuriyet University, 06532 Sivas, Turkey; b Department of Physics, Faculty of Sciences, Erciyes University, 38039 Kayseri, Turkey; c Department of Chemistry, Faculty of Science and Art, Gaziosmanpasa University, 60240 Tokat, Turkey; d Departamento Química Física y Analítica, Facultad de Química, Universidad Oviedo, C/ Julián Clavería, 8, 33006 Oviedo (Asturias), Spain

**Keywords:** crystal structure, cynarinin A, *Centaurea polypodiifolia*, theoretical investigation, CNDO, PM3, HOMO, LUMO

## Abstract

In the mol­ecule of title compound, C_15_H_20_O_6_, also known as cynarinin A, the cyclo­pentane ring having twist conformation and a γ-lactone ring assuming an envelope conformation are *trans*- and *cis*-fused, respectively, to a cyclo­heptane ring adopting a twist-chair conformation. In the crystal, O—H⋯O hydrogen bonds link neighbouring mol­ecules, forming a three-dimensional network. Theoretical calculations of the mol­ecular structure using the *CNDO* approximation and *MOPAC PM3* geometry optimization are in satisfactory agreement with the results of the X-ray structure analysis.

## Abstract 2

The cyclo­pentane ring displays a twist conformation and the γ-lactone ring has an envelope conformation while the cyclo­heptane ring adopts a twist-chair conformation. O—H⋯O hydrogen bonds link the mol­ecules, forming a three-dimensional network. A comparison between the structural parameters obtained by X-ray structure analysis and theoretical calculations give a satisfactory agreement.

## Chemical context   

The genus *Centaurea* belongs to the asteraceae family and consists of more than seven hundred species throughout the world. One hundred and ninety species are found in Turkey, one hundred of which are endemic (Davis *et al.*, 1988 ▸). *Centaurea* species contain acetyl­enic compounds (Christensen & Lam, 1990 ▸), flavonoids (Gulcemal *et al.*, 2010 ▸; Kubacey *et al.*, 2012 ▸; Khalfallah *et al.*, 2012 ▸; Forgo *et al.*, 2012 ▸) and sesquiterpene lactones (Bruno *et al.*, 1996 ▸; Koukoulitsa *et al.*, 2002 ▸; Janackovic *et al.*, 2004 ▸; Bensouici *et al.*, 2012 ▸), and display anti­cancer (Chicca *et al.*, 2011 ▸; Csapi *et al.*, 2010 ▸), anti­microbial, and anti-oxidant activities (Uysal *et al.*, 2013 ▸; Politeo *et al.*, 2012 ▸; Djeddi *et al.*, 2011 ▸). Sesquiterpene lactones (SLs) are a class of plant secondary metabolites of lipophilic character. SLs exhibit diverse biological activities such as anti-inflammatory, anti-ulcer, anti­bacterial, anti­viral, anti­fungal, and cytotoxic activity, and have an influence on the central nervous system and cardiovascular system (Yeşilada *et al.*, 1995 ▸). As a contribution to this research field, the X-ray crystal structure of the title compound, also known as cynarinin A (Kamanzi *et al.*, 1983 ▸), is reported herein.
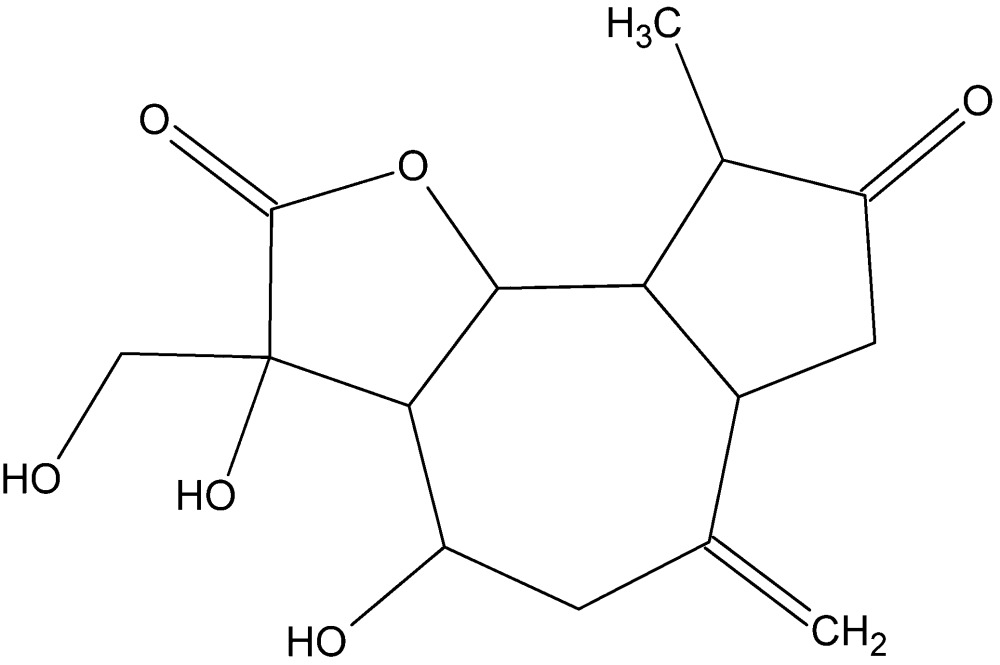


## Structural commentary   

**Figure 1:**
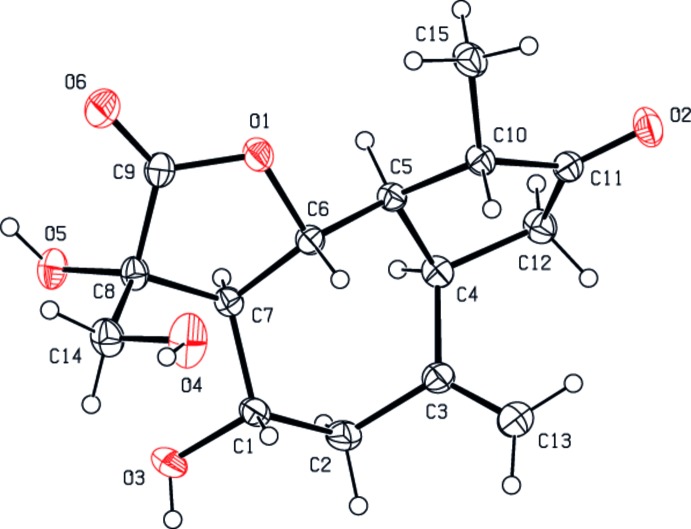
The mol­ecular structure of the title compound, with displacement ellipsoids drawn at the 50% probability level.

The title compound contains a cyclo­pentane ring and a γ-lactone ring *trans-* and *cis*-fused, respectively, to a cyclo­heptane ring (Fig. 1 ▸). The relative configurations at the asymmetric centres are C1(*S*), C4(*R*), C5(*R*), C6(*R*), C7(*R*), C8(*R*) and C10(*S*). The cyclo­pentane ring (C4/C5/C10–C12) is in a twist conformation about the C4—C5 bond with puckering parameters *Q* = 0.340 (3) Å and φ = 21.3 (4)°. The γ-lactone ring (O1/C6–C9) has an envelope conformation, with C7 at the flap [puckering parameters: *Q* = 0.271 (2) Å, φ = 259.0 (5)°]. The cyclo­heptane ring has a twist-chair conformation [puckering parameters: *Q*2 = 0.534 (2) Å, φ2 = 34.5 (3)°; Q3 = 0.650 (2) Å, φ3 = 191.5 (2)° and *Q*_T_ = 0.841 (2) Å]. The pseudo-diad axis bis­ects the C1—C2 bond and passes through atom C5. All bond lengths and angles are unexceptional and comparable with those reported for a similar compound (Swamy *et al.*, 2005 ▸).

## Supra­molecular features   

**Figure 2:**
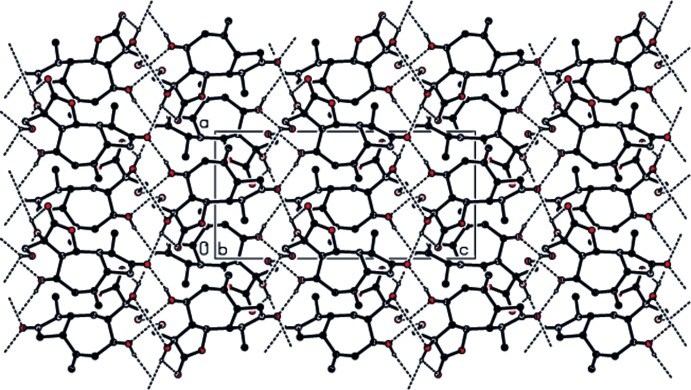
The crystal packing of the title compound, viewed down the *b* axis, showing the three-dimensional hydrogen-bonding network (dashed lines).

In the crystal, neighbouring mol­ecules are connected by O—H⋯O hydrogen bonds (Table 1 ▸; Fig. 2 ▸), forming a three dimensional network.

## Theoretical calculations   

**Figure 3:**
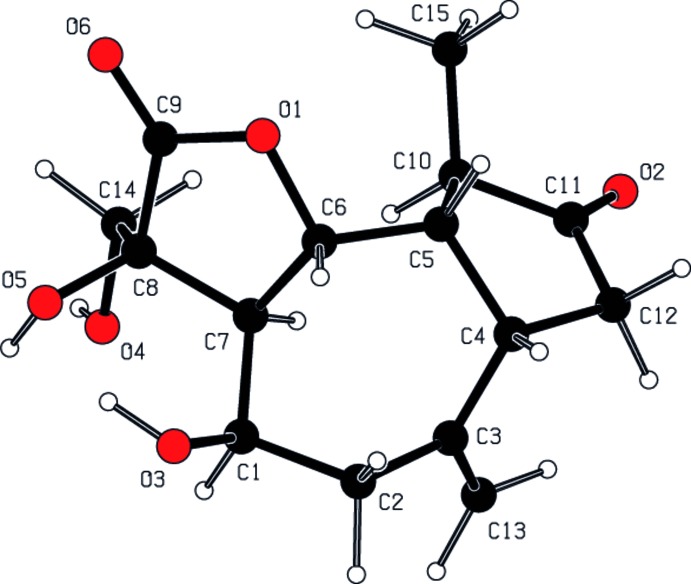
Spatial view of the mol­ecule of the title compound calculated using the *PM3* method.

According to the results of a quantum mechanical calculation using the *CNDO* approximation (Pople *et al.*, 1970 ▸), the charges at atoms O1, O2, O3, O4, O5 and O6 are −0.270, −0.241, −0.261, −0.255, −0.243 and −0.268 e^−^, respectively. The total energy and dipole moment of the title mol­ecule are −6339.85 eV and 3.211 Debye. The HOMO and LUMO energy levels are −12.5301 and 3.7741 eV, respectively. In addition, a geometrical optimization calculation of the title compound was performed using *MOPAC PM3* (Stewart, 1985 ▸). The spatial disposition of the atoms of the title mol­ecule calculated with *PM3* is shown in Fig. 3 ▸. The net charges at atoms O1, O2, O3, O4, O5 and O6 are −0.225, −0.304, −0.340, −0.318, −0.287 and −0.307e^−^, respectively. The total energy and dipole moment of the title mol­ecule are −3848.31 eV and 3.305 Debye. The HOMO and LUMO energy levels are −10.3738 and 0.5350 eV, respectively. In the calculations, the mol­ecule was assumed to be isolated and in an absolute vacuum therefore resulting in calculated bond lengths, bond angles and torsion angles that are greater than those observed experimentally. The *PM3* method gives the lowest values for the HOMO and LUMO energy levels and the dipole moment.

## Synthesis and crystallization   

*Centaurea polypodiifolia* Boiss. (1.0 kg) was extracted with methanol (3 × 5L), filtered, and the solvent removed *in vacuo* to obtain the crude material which was dissolved in water (333 K) and extracted with ethyl acetate. The organic phase was separated by separator funnel and the solvent was removed by reduced pressure to yield the extract (10 g). The extract was subjected to silica gel (60, GF_254_) column chromatography (2.5 cm × 60 cm). A hexa­ne/ethyl acetate mixture (6:4 *v*/*v*) was used as eluent. 24 fractions of 250 mL were collected. After checking by thin layer chromatography, 6–8 fractions were combined and crystallized in methanol to give suitable crystals of the title compound on slow evaporation of the solvent (yield: 10 mg). ^13^C NMR (150 MHz, DMSO-*d*_6_) δ 219.04 (C3), 178.88 (C12), 145.62 (C10), 113.64 (C14), 81.65 (C6), 78.36 (C11), 69.19 (C8), 63.68 (C13), 55.76 (C7), 51.31 (C5), 48.58 (C9), 46.91 (C4), 43.23 (C2), 39.66 (C1), 14.83 (C15). ^1^H NMR (600 MHz, DMSO-*d*_6_) δ 5.41 (*s*, 1H, 11-OH), 5.20 (*t*, 1H, *J =* 4.62 Hz 13-OH), 4.94 (*s*, 1H, H14a), 4.78 (*d*, 1H, *J* = 6.09 Hz, 8-OH), 4.63 (*s*, 1H, H14b), 4.04–3.93 (*m*, 3H, H6, H8 and H13a), 3.51 (*dd*, 1H, *J* = 9.78, 4.79 Hz, H13b), 3.07 (*dt*, 1H, *J* = 12.47, 4.06 Hz, H1), 2.67 (*dd*,1H, *J* = 12.28, 5.50 Hz, H9a), 2.51 (*dd*, 1H, *J* = 18.66, 8.97 Hz, H2a), 2.45 (*t*, 1H, *J* = 10.11 Hz, H7), 2.33 (*dd*, 1H, *J* = 18.66, 4.26 Hz, H2b), 2.21–2.14 (*m*, 2H, H4 and H5), 2.13–2.07 (*m*, 1H, H9b), 1.05 (d, 3H, *J* = 6.38 Hz, H15).

## Refinement   

Crystal data, data collection and structure refinement details are summarized in Table 2 ▸. H atoms bound to oxygen atoms were found in a difference Fourier map and allowed to ride on their parent atoms, with O—H = 0.82 Å and with *U*_iso_ = 1.5 *U*_eq_(O). H atoms bound to carbon atoms were placed in idealized positions and allowed to ride on their parent atoms, with C—H = 0.93–0.98 Å, and with *U*_iso_ = 1.2 *U*_eq_(C). One outlier (1 0 1) was omitted in the last cycles of refinement.

## Supplementary Material

Crystal structure: contains datablock(s) global, I. DOI: 10.1107/S2056989015019623/rz5172sup1.cif

Structure factors: contains datablock(s) I. DOI: 10.1107/S2056989015019623/rz5172Isup2.hkl

Supporting information file. DOI: 10.1107/S2056989015019623/rz5172Isup3.cml

CCDC reference: 1048445

Additional supporting information:  crystallographic information; 3D view; checkCIF report

## Figures and Tables

**Table 1:** Hydrogen-bond geometry (Å, °)

*D*—H⋯*A*	*D*—H	H⋯*A*	*D*⋯*A*	*D*—H⋯*A*
O3—H3*O*⋯O2^i^	0.78 (5)	2.06 (4)	2.818 (3)	168 (4)
O4—H4*O*⋯O3^ii^	0.95 (5)	2.14 (5)	2.956 (3)	144 (4)
O4—H4*O*⋯O5^ii^	0.95 (5)	2.45 (5)	3.156 (3)	132 (4)
O5—H5*O*⋯O6	0.90 (4)	2.45 (4)	2.877 (3)	109 (3)
O5—H5*O*⋯O2^iii^	0.90 (4)	2.22 (4)	3.096 (2)	164 (4)

Symmetry codes: (i) 
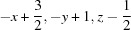
; (ii) 
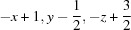
; (iii) 
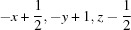
.

**Table 2:** Experimental details

Crystal data	
Chemical formula	C_15_H_20_O_6_
*M*_r_	296.31
Crystal system, space group	Orthorhombic, *P*2_1_2_1_2_1_
Temperature (K)	293
*a*, *b*, *c* (Å)	8.1980 (1), 10.0290 (2), 16.7720 (3)
*V* (Å^3^)	1378.96 (4)
*Z*	4
Radiation type	Cu *K*α
μ (mm^−1^)	0.92
Crystal size (mm)	0.65 × 0.47 × 0.30

Data collection	
Diffractometer	Agilent Xcalibur Ruby Gemini
Absorption correction	Multi-scan (*CrysAlis PRO*; Agilent, 2013 ▸)
*T*_min_, *T*_max_	0.773, 1.000
No. of measured, independent and observed [*I* > 2σ(*I*)] reflections	12778, 2623, 2502
*R*_int_	0.040
(sin θ/λ)_max_ (Å^−1^)	0.613

Refinement	
*R*[*F* ^2^ > 2σ(*F* ^2^)], *wR*(*F* ^2^), *S*	0.035, 0.096, 1.05
No. of reflections	2623
No. of parameters	200
Δρ_max_, Δρ_min_ (e Å^−3^)	0.27, −0.17
Absolute structure	Flack (1983 ▸), 1073 Friedel pairs
Absolute structure parameter	−0.09 (9)

Computer programs: *CrysAlis PRO* (Agilent, 2013 ▸), *SHELXS97* and *SHELXL97* (Sheldrick, 2008 ▸), *ORTEP-3 for Windows* (Farrugia, 2012 ▸) and *PLATON* (Spek, 2009 ▸).

## supplementary crystallographic information

### Crystal data

**Table:**

C_15_H_20_O_6_	*F*(000) = 632
*M**_r_* = 296.31	*D*_x_ = 1.427 Mg m^−^^3^
Orthorhombic, *P*2_1_2_1_2_1_	Cu *K*α radiation, λ = 1.54184 Å
Hall symbol: P 2ac 2ab	Cell parameters from 7099 reflections
*a* = 8.1980 (1) Å	θ = 4.4–70.5°
*b* = 10.0290 (2) Å	µ = 0.92 mm^−^^1^
*c* = 16.7720 (3) Å	*T* = 293 K
*V* = 1378.96 (4) Å^3^	Prism, colourless
*Z* = 4	0.65 × 0.47 × 0.30 mm

### Data collection

**Table:**

Agilent Xcalibur Ruby Gemini diffractometer	2623 independent reflections
Radiation source: Enhance (Cu) X-ray Source	2502 reflections with *I* > 2σ(*I*)
Graphite monochromator	*R*_int_ = 0.040
Detector resolution: 10.2673 pixels mm^-1^	θ_max_ = 70.9°, θ_min_ = 5.1°
ω scans	*h* = −10→7
Absorption correction: multi-scan (*CrysAlis PRO*; Agilent, 2013)	*k* = −12→12
*T*_min_ = 0.773, *T*_max_ = 1.000	*l* = −20→20
12778 measured reflections	

### Refinement

**Table:**

Refinement on *F*^2^	*w* = 1/[σ^2^(*F*_o_^2^) + (0.0594*P*)^2^ + 0.2045*P*] where *P* = (*F*_o_^2^ + 2*F*_c_^2^)/3
Least-squares matrix: full	(Δ/σ)_max_ < 0.001
*R*[*F*^2^ > 2σ(*F*^2^)] = 0.035	Δρ_max_ = 0.27 e Å^−^^3^
*wR*(*F*^2^) = 0.096	Δρ_min_ = −0.17 e Å^−^^3^
*S* = 1.05	Extinction correction: *SHELXL97* (Sheldrick, 2008), FC^*^=KFC[1+0.001XFC^2^Λ^3^/SIN(2Θ)]^-1/4^
2623 reflections	Extinction coefficient: 0.0184 (14)
200 parameters	Absolute structure: Flack (1983), 1073 Friedel pairs
0 restraints	Absolute structure parameter: −0.09 (9)
Hydrogen site location: mixed	

### Special details

**Table:**

Geometry. Bond distances, angles *etc*. have been calculated using the rounded fractional coordinates. All su's are estimated from the variances of the (full) variance-covariance matrix. The cell e.s.d.'s are taken into account in the estimation of distances, angles and torsion angles
Refinement. Refinement on *F*^2^ for ALL reflections except those flagged by the user for potential systematic errors. Weighted *R*-factors *wR* and all goodnesses of fit *S* are based on *F*^2^, conventional *R*-factors *R* are based on *F*, with *F* set to zero for negative *F*^2^. The observed criterion of *F*^2^ > σ(*F*^2^) is used only for calculating -*R*-factor-obs *etc*. and is not relevant to the choice of reflections for refinement. *R*-factors based on *F*^2^ are statistically about twice as large as those based on *F*, and *R*-factors based on ALL data will be even larger.

### Fractional atomic coordinates and isotropic or equivalent isotropic displacement parameters (Å^2^)

**Table:**

	*x*	*y*	*z*	*U*_iso_*/*U*_eq_	
O1	0.2767 (2)	0.42340 (18)	0.94867 (10)	0.0363 (5)	
O2	0.5458 (2)	0.3191 (2)	1.23883 (10)	0.0417 (6)	
O3	0.6704 (3)	0.7021 (2)	0.83279 (12)	0.0430 (6)	
O4	0.4798 (3)	0.3608 (2)	0.79632 (13)	0.0583 (8)	
O5	0.2968 (2)	0.68723 (17)	0.81640 (11)	0.0385 (5)	
O6	0.0874 (2)	0.4674 (2)	0.85721 (12)	0.0526 (7)	
C1	0.6584 (3)	0.5976 (2)	0.89063 (13)	0.0298 (7)	
C2	0.7676 (3)	0.6329 (3)	0.96189 (15)	0.0371 (7)	
C3	0.7539 (3)	0.5438 (2)	1.03466 (14)	0.0324 (7)	
C4	0.6038 (3)	0.5670 (2)	1.08525 (13)	0.0294 (7)	
C5	0.4523 (3)	0.4859 (2)	1.05600 (13)	0.0259 (6)	
C6	0.4448 (3)	0.4643 (2)	0.96656 (13)	0.0266 (6)	
C7	0.4782 (3)	0.5846 (2)	0.91215 (13)	0.0255 (6)	
C8	0.3588 (3)	0.5632 (2)	0.84275 (13)	0.0284 (6)	
C9	0.2237 (3)	0.4813 (2)	0.88134 (14)	0.0331 (7)	
C10	0.4595 (3)	0.3538 (2)	1.10233 (13)	0.0280 (6)	
C11	0.5437 (3)	0.3898 (2)	1.17991 (13)	0.0301 (7)	
C12	0.6183 (3)	0.5262 (3)	1.17351 (14)	0.0347 (7)	
C13	0.8685 (3)	0.4549 (3)	1.05183 (18)	0.0476 (9)	
C14	0.4213 (4)	0.4848 (3)	0.77071 (15)	0.0384 (8)	
C15	0.3005 (3)	0.2790 (3)	1.11343 (15)	0.0389 (8)	
H1	0.69630	0.51390	0.86680	0.0360*	
H2A	0.88010	0.63170	0.94400	0.0450*	
H2B	0.74270	0.72350	0.97810	0.0450*	
H3O	0.755 (6)	0.703 (4)	0.812 (2)	0.0650*	
H4	0.57660	0.66200	1.08300	0.0350*	
H4O	0.480 (6)	0.298 (5)	0.754 (3)	0.0880*	
H5	0.35370	0.53380	1.07230	0.0310*	
H5O	0.200 (5)	0.668 (4)	0.794 (2)	0.0580*	
H6	0.51910	0.39170	0.95210	0.0320*	
H7	0.44530	0.66550	0.94070	0.0310*	
H10	0.53330	0.29460	1.07310	0.0340*	
H12A	0.73190	0.52400	1.18980	0.0420*	
H12B	0.56050	0.58900	1.20720	0.0420*	
H13A	0.85900	0.40230	1.09730	0.0570*	
H13B	0.95820	0.44510	1.01840	0.0570*	
H14A	0.33360	0.47210	0.73260	0.0460*	
H14B	0.50810	0.53410	0.74470	0.0460*	
H15A	0.25390	0.25940	1.06220	0.0580*	
H15B	0.32090	0.19720	1.14150	0.0580*	
H15C	0.22600	0.33280	1.14360	0.0580*	

### Atomic displacement parameters (Å^2^)

**Table:**

	*U*^11^	*U*^22^	*U*^33^	*U*^12^	*U*^13^	*U*^23^
O1	0.0354 (9)	0.0441 (9)	0.0295 (8)	−0.0134 (7)	−0.0076 (7)	0.0066 (8)
O2	0.0504 (11)	0.0463 (10)	0.0285 (9)	−0.0056 (9)	−0.0076 (8)	0.0089 (8)
O3	0.0399 (10)	0.0489 (11)	0.0403 (10)	−0.0025 (9)	0.0115 (8)	0.0163 (9)
O4	0.0839 (17)	0.0463 (12)	0.0448 (11)	0.0247 (12)	−0.0133 (11)	−0.0176 (9)
O5	0.0447 (10)	0.0302 (8)	0.0407 (9)	0.0053 (8)	−0.0094 (8)	0.0039 (8)
O6	0.0391 (11)	0.0711 (15)	0.0477 (11)	−0.0126 (10)	−0.0136 (9)	0.0099 (10)
C1	0.0314 (12)	0.0314 (12)	0.0266 (10)	−0.0001 (10)	0.0055 (9)	0.0032 (10)
C2	0.0306 (12)	0.0426 (13)	0.0380 (13)	−0.0097 (11)	0.0022 (10)	0.0041 (11)
C3	0.0280 (12)	0.0377 (12)	0.0316 (12)	−0.0081 (10)	−0.0029 (9)	0.0002 (10)
C4	0.0336 (12)	0.0265 (11)	0.0280 (11)	−0.0041 (9)	−0.0003 (10)	−0.0019 (9)
C5	0.0263 (10)	0.0268 (10)	0.0245 (10)	−0.0014 (8)	0.0010 (9)	−0.0005 (8)
C6	0.0271 (11)	0.0273 (11)	0.0254 (11)	−0.0027 (9)	−0.0011 (8)	0.0001 (8)
C7	0.0294 (11)	0.0239 (10)	0.0232 (10)	0.0018 (9)	0.0020 (9)	−0.0017 (8)
C8	0.0341 (12)	0.0255 (11)	0.0256 (10)	0.0043 (9)	−0.0024 (9)	−0.0004 (9)
C9	0.0339 (13)	0.0363 (12)	0.0290 (11)	−0.0009 (10)	−0.0050 (10)	−0.0016 (10)
C10	0.0322 (11)	0.0280 (11)	0.0239 (10)	−0.0019 (9)	0.0002 (9)	−0.0003 (8)
C11	0.0285 (11)	0.0360 (12)	0.0257 (11)	0.0012 (10)	−0.0001 (10)	−0.0007 (9)
C12	0.0390 (13)	0.0385 (13)	0.0266 (11)	−0.0052 (11)	−0.0022 (10)	−0.0040 (10)
C13	0.0364 (14)	0.0650 (19)	0.0413 (14)	0.0038 (13)	−0.0002 (12)	0.0033 (14)
C14	0.0478 (15)	0.0404 (14)	0.0269 (11)	0.0048 (12)	−0.0004 (11)	−0.0054 (10)
C15	0.0414 (14)	0.0425 (14)	0.0328 (12)	−0.0135 (12)	−0.0028 (11)	0.0052 (11)

### Geometric parameters (Å, º)

**Table:**

O1—C6	1.469 (3)	C8—C9	1.523 (3)
O1—C9	1.342 (3)	C10—C11	1.517 (3)
O2—C11	1.216 (3)	C10—C15	1.515 (4)
O3—C1	1.432 (3)	C11—C12	1.502 (4)
O4—C14	1.400 (4)	C1—H1	0.9800
O5—C8	1.415 (3)	C2—H2A	0.9700
O6—C9	1.197 (3)	C2—H2B	0.9700
O3—H3O	0.78 (5)	C4—H4	0.9800
O4—H4O	0.95 (5)	C5—H5	0.9800
O5—H5O	0.90 (4)	C6—H6	0.9800
C1—C2	1.535 (3)	C7—H7	0.9800
C1—C7	1.526 (3)	C10—H10	0.9800
C2—C3	1.517 (4)	C12—H12A	0.9700
C3—C4	1.513 (3)	C12—H12B	0.9700
C3—C13	1.327 (4)	C13—H13A	0.9300
C4—C5	1.564 (3)	C13—H13B	0.9300
C4—C12	1.540 (3)	C14—H14A	0.9700
C5—C6	1.517 (3)	C14—H14B	0.9700
C5—C10	1.537 (3)	C15—H15A	0.9600
C6—C7	1.537 (3)	C15—H15B	0.9600
C7—C8	1.536 (3)	C15—H15C	0.9600
C8—C14	1.530 (3)		
			
C6—O1—C9	110.78 (17)	C2—C1—H1	109.00
C1—O3—H3O	112 (3)	C7—C1—H1	109.00
C14—O4—H4O	111 (3)	C1—C2—H2A	108.00
C8—O5—H5O	105 (3)	C1—C2—H2B	108.00
O3—C1—C7	106.82 (19)	C3—C2—H2A	108.00
O3—C1—C2	108.59 (19)	C3—C2—H2B	108.00
C2—C1—C7	113.59 (19)	H2A—C2—H2B	107.00
C1—C2—C3	116.6 (2)	C3—C4—H4	108.00
C2—C3—C4	114.90 (19)	C5—C4—H4	108.00
C4—C3—C13	123.9 (2)	C12—C4—H4	108.00
C2—C3—C13	121.2 (2)	C4—C5—H5	108.00
C5—C4—C12	102.98 (18)	C6—C5—H5	108.00
C3—C4—C5	112.96 (18)	C10—C5—H5	108.00
C3—C4—C12	115.8 (2)	O1—C6—H6	109.00
C4—C5—C6	114.63 (19)	C5—C6—H6	109.00
C4—C5—C10	105.03 (18)	C7—C6—H6	109.00
C6—C5—C10	112.24 (17)	C1—C7—H7	108.00
O1—C6—C5	106.26 (18)	C6—C7—H7	108.00
O1—C6—C7	105.37 (18)	C8—C7—H7	108.00
C5—C6—C7	117.89 (17)	C5—C10—H10	107.00
C1—C7—C8	116.72 (19)	C11—C10—H10	107.00
C1—C7—C6	112.32 (18)	C15—C10—H10	107.00
C6—C7—C8	103.10 (17)	C4—C12—H12A	111.00
O5—C8—C7	110.05 (17)	C4—C12—H12B	110.00
O5—C8—C9	110.22 (19)	C11—C12—H12A	110.00
C9—C8—C14	107.58 (19)	C11—C12—H12B	111.00
C7—C8—C14	117.2 (2)	H12A—C12—H12B	109.00
O5—C8—C14	109.00 (19)	C3—C13—H13A	120.00
C7—C8—C9	102.52 (18)	C3—C13—H13B	120.00
O1—C9—O6	122.5 (2)	H13A—C13—H13B	120.00
O1—C9—C8	110.8 (2)	O4—C14—H14A	110.00
O6—C9—C8	126.7 (2)	O4—C14—H14B	110.00
C5—C10—C11	104.24 (17)	C8—C14—H14A	110.00
C5—C10—C15	117.1 (2)	C8—C14—H14B	110.00
C11—C10—C15	113.83 (19)	H14A—C14—H14B	108.00
O2—C11—C12	125.7 (2)	C10—C15—H15A	109.00
O2—C11—C10	124.4 (2)	C10—C15—H15B	109.00
C10—C11—C12	109.92 (18)	C10—C15—H15C	110.00
C4—C12—C11	106.21 (19)	H15A—C15—H15B	109.00
O4—C14—C8	109.2 (2)	H15A—C15—H15C	109.00
O3—C1—H1	109.00	H15B—C15—H15C	109.00
			
C6—O1—C9—O6	−176.4 (2)	C6—C5—C10—C15	−79.1 (3)
C6—O1—C9—C8	3.7 (2)	C4—C5—C6—O1	−163.79 (17)
C9—O1—C6—C5	139.66 (18)	O1—C6—C7—C1	−151.38 (17)
C9—O1—C6—C7	13.8 (2)	C5—C6—C7—C8	−143.2 (2)
O3—C1—C2—C3	171.8 (2)	O1—C6—C7—C8	−24.9 (2)
O3—C1—C7—C8	54.4 (2)	C5—C6—C7—C1	90.3 (2)
C7—C1—C2—C3	53.2 (3)	C1—C7—C8—C14	32.2 (3)
O3—C1—C7—C6	173.17 (17)	C6—C7—C8—O5	143.33 (18)
C2—C1—C7—C6	−67.1 (2)	C6—C7—C8—C9	26.1 (2)
C2—C1—C7—C8	174.11 (19)	C6—C7—C8—C14	−91.4 (2)
C1—C2—C3—C13	104.8 (3)	C1—C7—C8—C9	149.70 (18)
C1—C2—C3—C4	−76.4 (3)	C1—C7—C8—O5	−93.1 (2)
C2—C3—C4—C5	86.5 (2)	O5—C8—C9—O6	43.5 (3)
C2—C3—C4—C12	−155.1 (2)	O5—C8—C9—O1	−136.56 (19)
C13—C3—C4—C5	−94.8 (3)	C14—C8—C9—O1	104.7 (2)
C13—C3—C4—C12	23.7 (3)	C14—C8—C9—O6	−75.2 (3)
C12—C4—C5—C10	−34.5 (2)	O5—C8—C14—O4	−178.8 (2)
C12—C4—C5—C6	−158.11 (19)	C7—C8—C14—O4	55.4 (3)
C5—C4—C12—C11	26.5 (2)	C9—C8—C14—O4	−59.3 (3)
C3—C4—C5—C10	91.2 (2)	C7—C8—C9—O1	−19.4 (2)
C3—C4—C5—C6	−32.5 (2)	C7—C8—C9—O6	160.6 (2)
C3—C4—C12—C11	−97.3 (2)	C5—C10—C11—O2	165.6 (2)
C10—C5—C6—C7	−165.7 (2)	C5—C10—C11—C12	−12.6 (3)
C4—C5—C10—C15	155.79 (19)	C15—C10—C11—O2	36.8 (3)
C6—C5—C10—C11	154.2 (2)	C15—C10—C11—C12	−141.4 (2)
C4—C5—C10—C11	29.1 (2)	O2—C11—C12—C4	172.8 (2)
C4—C5—C6—C7	−46.0 (3)	C10—C11—C12—C4	−9.1 (3)
C10—C5—C6—O1	76.5 (2)		

### Hydrogen-bond geometry (Å, º)

**Table:**

*D*—H···*A*	*D*—H	H···*A*	*D*···*A*	*D*—H···*A*
O3—H3*O*···O2^i^	0.78 (5)	2.06 (4)	2.818 (3)	168 (4)
O4—H4*O*···O3^ii^	0.95 (5)	2.14 (5)	2.956 (3)	144 (4)
O4—H4*O*···O5^ii^	0.95 (5)	2.45 (5)	3.156 (3)	132 (4)
O5—H5*O*···O6	0.90 (4)	2.45 (4)	2.877 (3)	109 (3)
O5—H5*O*···O2^iii^	0.90 (4)	2.22 (4)	3.096 (2)	164 (4)

Symmetry codes: (i) −*x*+3/2, −*y*+1, *z*−1/2; (ii) −*x*+1, *y*−1/2, −*z*+3/2; (iii) −*x*+1/2, −*y*+1, *z*−1/2.
